# Targeted dream incubation at a distance: the development of a remote and sensor-free tool for incubating hypnagogic dreams and mind-wandering

**DOI:** 10.3389/frsle.2024.1258345

**Published:** 2024-05-28

**Authors:** Lucas Bellaiche, Adam Haar Horowitz, Mason McClay, Ryan Bottary, Dan Denis, Christina Chen, Pattie Maes, Paul Seli

**Affiliations:** ^1^Department of Psychology and Neuroscience, Duke University, Durham, NC, United States; ^2^MIT Media Lab, Massachusetts Institute of Technology, Cambridge, MA, United States; ^3^Center for Sleep and Cognition, Department of Psychiatry, Beth Israel Deaconess Medical Center, Boston, MA, United States; ^4^Outer Coast College, Sitka, AK, United States; ^5^Department of Psychology, University of California, Los Angeles, Los Angeles, CA, United States; ^6^Institute for Graduate Clinical Psychology, Widener University, Chester, PA, United States; ^7^Department of Psychology, University of York, York, United Kingdom

**Keywords:** hypnagogia, hypnagogic dreams, mind-wandering, freely moving thought, dream incubation

## Abstract

Hypnagogia—the transitional state between wakefulness and sleep—is marked by “hypnagogic dreams,” during which our brains tend to forge connections among concepts that are otherwise unrelated. This process of creating novel associations during hypnagogic dreams is said to contribute to enhancing creativity, learning, and memory. Recently, researchers have proposed that mind-wandering—a form of spontaneous thought that is freely moving and characterized by transitioning thought content—might be subserved by processes similar to those engaged during hypnagogia, and may serve similar creative functions. However, to date, the relationship between hypnagogia and mind-wandering remains poorly understood, which is likely due in part to the fact that research into hypnagogia requires time-consuming, cumbersome, and costly polysomnography. In light of this, the present study had two primary aims: first, to test a novel tool—called Dormio Light—for cueing and indexing hypnagogic dream content in a cost- and time-effective manner, with the ability for remote administration; second, to use this tool to examine any relations between hypnagogic dreams and mind-wandering (defined as “freely moving thought”). Participants (*N* = 80, with 34 females) completed a task in which our tool prompted them to engage in hypnagogia and, separately, mind-wandering, with instructions to think about a common everyday object (Tree or Fork) while experiencing these cognitive states. Following each state, participants reported thought content and completed phenomenological questionnaires. Providing an initial validation of our tool, we successfully cued hypnagogic and mind-wandering thought content that was specific to our cues (e.g., Tree), with our incubation-rate results comparable to those found in laboratory-based studies. Further, we found evidence for some phenomenological differences between hypnagogia and mind-wandering reports. Our study offers a novel, cost- and time-effective tool with which to remotely cue and index hypnagogia and mind-wandering, and sheds light on the relationship between hypnagogia and mind-wandering, thereby providing future directions for research into these two cognitive states.

## Introduction

In recent years, there has been a surge of psychological research into different states of human consciousness. Here, we concentrate on two such manifestations of consciousness: hypnagogia and freely moving thought (a particular type of mind-wandering). Hypnagogia, also known as Stage N1, refers to the transitional state from wakefulness to sleep, where one may experience a diverse array of sensory phenomena, including auditory or visual hallucinations, lucid dreams (Mota-Rolim et al., 2015), or even a sense of falling or floating. Hypnagogia is characterized by spontaneous dreams—“hypnagogic dreams”—during which our brains tend to forge novel connections between otherwise semantically disparate concepts (Schacter, 1976; Ghibellini and Meier, 2023). On the other hand, freely moving thought (FMT; a type of mind-wandering) refers to a cognitive state, experienced during waking life, wherein people's thoughts make frequent transitions across semantically unrelated content (Mills et al., 2018). The present study had two primary aims: Firstly, to develop and validate an innovative tool that would allow for cuing and capturing thought content during hypnagogic and FMT states. Secondly, to identify and then compare and contrast the characteristics of thoughts individuals encounter within these two cognitive states.

### A new tool for cueing and capturing hypnagogic and mind-wandering thought content

Recently, researchers have shown increasing interest in *Targeted Dream Incubation* (TDI), a technique that involves the presentation of auditory cues during hypnagogia to introduce specific themes into people's hypnagogic dreams (Haar Horowitz et al., 2020). Much of the interest in TDI has stemmed from the potential of this technique to be utilized to enhance creative problem-solving and learning. Indeed, it has been speculated that by guiding the dreaming mind toward particular content, researchers might be able to facilitate the forging of novel connections between otherwise disparate concepts—a process critical to creativity (Haar Horowitz et al., 2023; see also Lacaux et al., 2021).

To provide a foundation for research on TDI in hypnagogia, Haar Horowitz et al. (2020) developed a novel TDI tool, Dormio, which is a wearable electronic glove that indexes heart rate, muscle flexion, and electrodermal activity to identify the onset of hypnagogia. Once a hypnagogic state is identified, Dormio can then be utilized to deliver auditory cues which influence dream content and later can prompt and record dream reports. Utilizing this tool, Haar Horowitz et al. (2023) recently found that TDI used to incubate dreams on a specific topic can significantly increase post-sleep creativity on tasks related to that topic. However, while it has been established that Dormio is effective in cueing and indexing hypnagogic dream content (Haar Horowitz et al., 2020, 2023), research implementing Dormio can be difficult to conduct. Indeed, the Dormio glove is custom-made and few devices exist; these sensitive devices can break, and at-home studies can suffer from delays. Moreover, collecting a large-data sample is limited by the production of hardware and lack of large-scale manufacturing of dream incubation devices.

Given the resource-demanding nature of using Dormio hardware, here, we sought to develop a modified version of Dormio that is software-based only (which we refer henceforth as “Dormio Light”) and would (a) remove the need for time-consuming, in-person procedures, (b) eliminate the requirement for hardware used to identify the onset of hypnagogia, (c) allow for remote cueing and indexing of hypnagogic dream content (e.g., via online data-collection platforms such as Prolific and Mechanical Turk), and (d) permit expedited data collection. To this end, we created Dormio Light, an online platform that induces specific dream content via TDI on a laptop's web browser. TDI incubates dream content using timed prompts that remind participants of their dream cue and prompt dream reports at appropriate times in the sleep cycle (see *Methods*). Crucially, because Dormio Light does not require hardware outside of a personal computer, this online platform permits researchers to use crowdsourcing websites to—for the first time in dream research to our knowledge—achieve large and same-day data collection. Given the methodological barriers (see Escourrou et al., 2000; Burgdorf et al., 2018; Topalidis et al., 2023) that constrain dream studies to small sample sizes (e.g., 50 participants in Haar Horowitz et al., 2023), such a development in remote dream research could provide important future research opportunities.

While the primary motivation behind the creation of Dormio Light was to streamline research into hypnagogia, it is important to consider the potential utility of this tool in cueing and subsequently incubating mind-wandering, specifically FMT. To our knowledge, the cueing of FMT has not yet been the subject of empirical investigation. Nonetheless, such cueing is of potential importance for a few reasons. Firstly, akin to research on targeted dream incubation, the cueing of FMT could unveil critical insights into the processes underlying FMT phenomenology, including its onset and flow. Indeed, one drawback of typical mind-wandering research, including that which uses experience-sampling methodologies, is the lack of identification of the “ignition point,” as raised by Smallwood (2013). In other words, “it is difficult to separate those processes that acted as the imperative event from the processes that are concerned with how those thoughts are sustained” (p. 521, 522). With a cueing procedure, as in the present study, this concern is largely eliminated by providing standardized “ignition points.” Additionally, Dormio Light could be deployed to minimally guide the content explored during FMT, potentially serving as a mechanism to foster creativity and problem-solving skills during these wakeful states.

### Phenomenological comparisons across hypnagogia and mind-wandering

Beyond developing a novel tool for guiding and capturing thoughts occurring during hypnagogia and periods of FMT, we were interested in examining the possible similarities and differences in thought content produced during these two states. On the one hand, there is reason to suspect some overlap in the content of thought across hypnagogia and FMT. Indeed, both states are characterized by cognitions that are relatively unconstrained and highly fluid (Perogamvros et al., 2017; Mills et al., 2018; Quercia et al., 2018; Andrillon et al., 2019), and it is therefore plausible that the thoughts engaged during these two states will have some commonalities. Moreover, recent research has found that both states are associated with enhanced creativity (Lacaux et al., 2021; Irving et al., 2022; Haar Horowitz et al., 2023)—presumably because the lack of constraint that is characteristic of these states allows for novel links to be drawn between different concepts—suggesting the possibility that there are similarities in thought content produced during each state. On the other hand, these two states are associated with distinct cognitive processes and experiences that should be expected to produce some differences in terms of thought content. Perhaps the most obvious difference in this respect is that, whereas hypnagogia occurs during a transitional period between wakefulness and sleep, FMT occur exclusively during wakefulness, when one's awareness of one's thoughts is presumably greater than during hypnagogia. Moreover, whereas hypnagogia often includes more dream-like or hallucinatory experiences (Schacter, 1976; Ghibellini and Meier, 2023), FMT do not appear to have such phenomenological characteristics.

Importantly, these two states likely exist on a continuum of cognitive control (as implied by the continuity hypothesis of dreaming; Schredl and Hofmann, 2003; see Sodré et al., 2023), with hypnagogia representing a state of reduced control and increased immersion in internally generated experiences, and FMT reflecting a state of reduced, but still present, control over thought content. However, to date, no research has directly compared the thought phenomenology across these two states. Thus, here, to shed light on the similarities and differences between thought content produced during hypnagogia and FMT, we indexed the characteristics of thoughts reported while participants experienced hypnagogia and, separately, FMT, via a thought-report questionnaire adopted from Smallwood et al. (2016) and Gross et al. (2020) that indexes several features of thought content and structure (e.g., Emotionality, Novelty, Topical Shifting, Meaningfulness). Given the exploratory nature of these comparisons, we do not report any specific hypotheses.

### The current study

Here, we used Dormio Light to provide timed prompts via the Targeted Dream Incubation (TDI) method to cue specific thought content for both hypnagogic dream states and FMT. Methodologically, we sought to validate our novel remote method by assessing incorporation rates of cued items (i.e., how many thought reports referenced the cued item) and compare them to similar in-person studies that utilized the Dormio glove (e.g., Haar Horowitz et al., 2020, 2023). In addition, we compared thought content—indexed via typed thought reports and responses to a thought-content questionnaire—to assess potential differences and similarities in thought profile across periods of hypnagogia and FMT.

## Methods

The following study was approved by Duke University Campus Institutional Review Board (2021–0422).

### Participants

Participants were recruited through Prolific, an online crowdsourcing platform that offers paid research studies to users worldwide. Eligibility criteria required participants to be at least 18 years old, fluent in English, residents of the United States, and have a minimum 90% approval rating from previous studies on Prolific. Additionally, participants needed to have completed at least 50 Prolific studies previously. For compatibility with the study's website, participants were also required to use Google Chrome as their web browser.

In total, we recruited 132 participants who met the Prolific requirements listed above. Given the exploratory nature of the study, we did not conduct any a priori power analyses, but we did aim to surpass sample sizes from previous cued-mind-wandering paradigms (e.g., McVay and Kane, 2013, reported between 57 and 67 participants in each of their experiments) and cued-hypnagogia studies (Haar Horowitz et al., 2020, 2023: 50 participants). However, given the novel remote methodology, we wanted to ensure that we analyzed data only from people who followed instructions completely and for whom the hypnagogic and FMT cueing worked effectively. Accordingly, we employed strict analysis inclusion criteria such that participants had to self-report (a) having stayed completely awake without any intervening sleep during the FMT task—which may be more common than expected, as reported in Tagliazucchi and Laufs (2014) and Andrillon et al. (2019)—and (b) having experienced some level of sleep (“fully” or “halfway”) during the hypnagogia task. Excluding data from participants who did not meet both of these stringent criteria, we report results from analyses examining data from 80 participants (*M*_*age*_ = 36.01, *SD*_*age*_ = 12.42; female = 34), which is above the target sample size and relatively high in power given the within-subjects design. We compensated participants $24.00 for an average experiment length of 2.6 hours.

### Dormio Light website

We guided participants to a website entitled “Dormio Light” for thought incubation, awakenings, and verbal reports. For interested readers, the Dormio Light website can be found at: https://christinatchen.github.io/dormio/timer.html. Participants completed both conditions (hypnagogic dreaming, FMT) separately in a randomized order via this website. Written instructions and pre-recorded video instructions in the Qualtrics survey prompted participants to enter, in the Dormio Light website, their Prolific ID, the object about which they were instructed to dream/mind-wander (randomly assigned by Qualtrics as either Fork or Tree), and to record audio messages in their own voices that the website would replay throughout the assigned condition. The self-recorded audio messages were (1) an incubation message (“Remember to think of a Fork/Tree”) intermittently played throughout the condition, and (2) a report message (“Tell me what is going through your mind”) that played four times across the condition prompting a verbal report of the participant's current thoughts. Research has indicated audio played during sleep in one's own voice can effectively incubate dream content (Castaldo and Holzman, 1967). Thus, here, we chose to test our tool using participants' own voices in order to make our tool as flexible as possible for at-home experimental use in the future.

**Figure 1:**
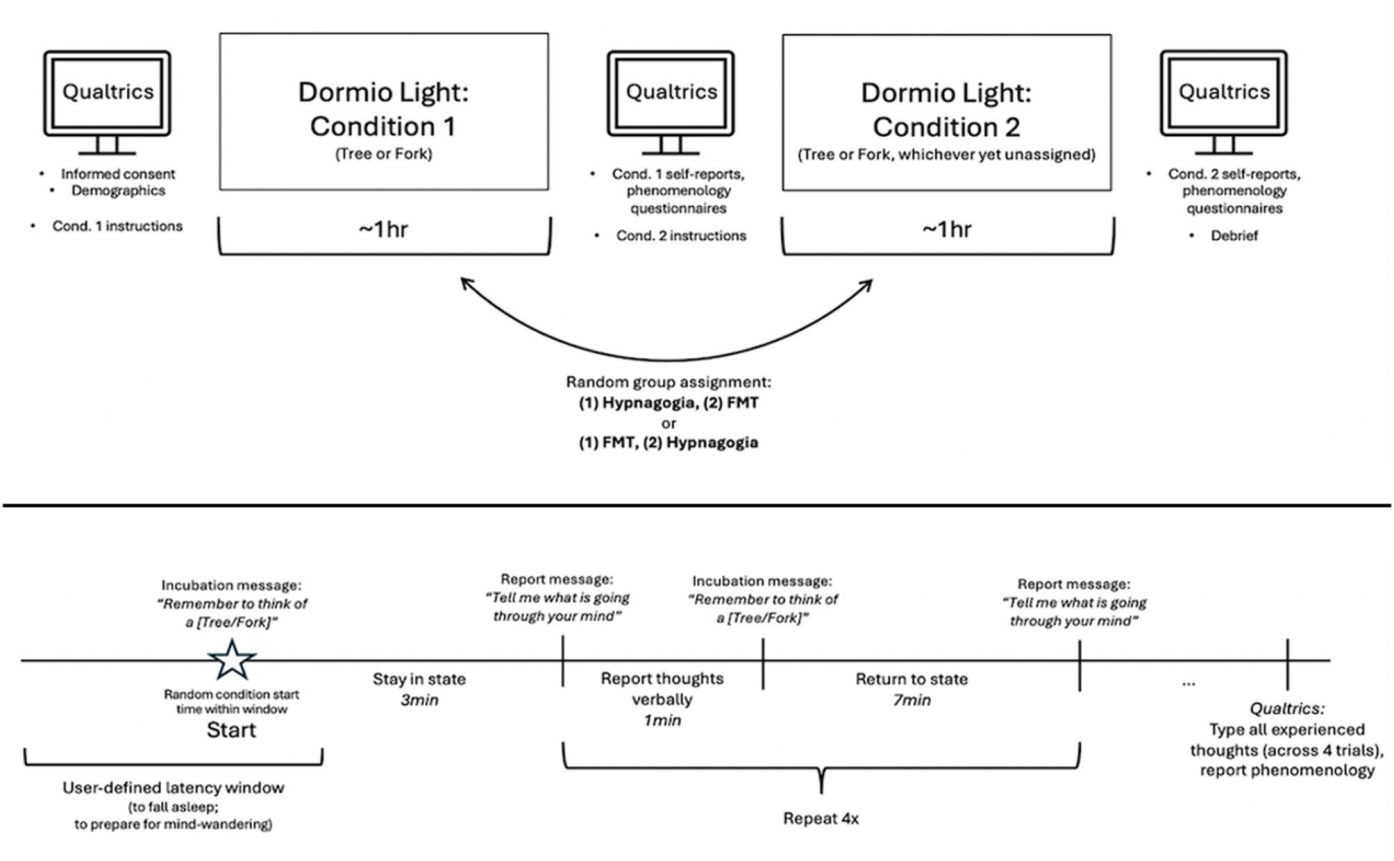
Study design. **(A)** Entire experiment procedure. **(B)** Procedure within each condition.

Additionally, we instructed participants to enter, in the Dormio Light website, a latency window of time to begin the condition (i.e., preparatory time to fall asleep into hypnagogia or to engage in FMT), after which the website would pick a random time within this window estimate. For instance, in the hypnagogia condition, a participant could enter that it typically takes him/her 10 to 15 min to fall asleep, and the Dormio Light website would present the incubation message after 13 min. This preparatory window of time was freely chosen by the participant given the individual variability that can exist regarding latency to sleep (Carskadon and Dement, 1982). The remainder of the settings were the same for all participants: to stay in hypnagogia/FMT for 3 min, record 4 rounds (i.e., trials) of entry into hypnagogia/FMT and report of thoughts, take 60 s to provide verbal reports as cued by their audio messages, and take 7 min to fall back into sleep/remain in a stable hypnagogia/FMT state following each awakening (see Figure 1). We chose these parameters for several reasons. First, though individual differences exist in sleep behavior, research suggests that hypnagogic dreaming is achieved rather rapidly after sleep onset. In addition, we believed that staying 3 min in each state provided adequate time for descriptive stories without a loss of memory during each of the 4 reports, which can occur if individuals enter N2 (Carr and Solomonova, 2019). Lastly, a short verbal report period allows for capture of cognitive content while mitigating the difficulty in falling back asleep which an increase in arousal during a longer period of awake report might create (Horner et al., 1997).

### Hypnagogia and FMT content reports

Immediately following each condition, participants typed their experienced thoughts across the four trials in a free-response question in Qualtrics, which allowed us to determine whether there were any differences between hypnagogia and FMT in *post-hoc* reports. We analyzed cue incorporation rates in these typed reports to ensure the prompting of cues via Dormio Light was effective for remote incubation, and to compare rates of incorporation between conditions within this remote experiment and with other previous in-lab methods of dream incubation cueing.

To examine the semantic properties of typed reports (which were cleaned to contain only text related to thought content^1^), we used the freely available “transformer” version of the Universal Sentence Encoder (USE), a text-embedding model designed to convert text into numerical vectors (Cer et al., 2018). Briefly, the USE uses pre-weighted layers, previously trained on an expansive textual database, to transform inputted sentences into 512-dimensional embedding vectors that account for words and their respective positions within each sentence. Then, we calculated cosine similarity between each sentence vector, yielding pairwise measures of textual similarity for all inputted sentences. As such, USE similarity serves as a proxy of word-level and sentence-level semantic relatedness for text. We employed the USE to derive sentence-embeddings for two measures of semantic structure in the FMT and hypnagogia reports: (1) prompt centrality, and (2) temporal coherence. We defined prompt centrality as the average similarity of every sentence in a given report to its target prompt. For instance, if a session's cued object was “Fork,” the similarity of each sentence in the report to the word “fork” would be derived. To control for the similarity between the incorporation of prompt words and similarity to the prompt word itself, we filtered all prompt words from the reports before we derived prompt centrality. Additionally, temporal coherence was defined as the similarity from sentence-to-sentence as a sliding window over a report. Specifically, we derived the similarity between sentence 1 and 2, then 2 and 3, so on and so forth. We calculated the temporal coherence score as the average sliding window similarity for an entire report, only for reports with two or more sentences. Both prompt centrality and temporal coherence were analyzed using linear mixed models. For all models, we determined the statistical significance of the regression model by performing a likelihood ratio test for the full model against a model which included all independent variables except for the effect of interest. All models included a random intercept for participant.

### Thought probes

We further assessed the content of participants' thoughts, both when in FMT and in hypnagogic sleep, using a modified version of the thought probe scales adapted from Smallwood et al. (2016) and Gross et al. (2020). The modified scale consists of a set of 13 questions measuring features of thoughts (Table 1).

**Table 1:** Thought probe scale.

**Item**	**Question**	**Response scale**
(1) Positive valence	The content of my thoughts was positive.	Completely disagree – Completely agree (1–5)
(2) Negative valence	The content of my thoughts was negative.	Completely disagree – Completely agree (1–5)
(3) Structure: images	My thoughts were in the form of images.	Completely disagree – Completely agree (1–5)
(4) Structure: words	My thoughts were in the form of words.	Completely disagree – Completely agree (1–5)
(5) Novelty	My thoughts were novel (that is, I've never experienced or thought them before).	Completely disagree – Completely agree (1–5)
(6) Freedom of thought flow	My thoughts were freely moving (i.e., I wasn't guiding them)	Completely disagree – Completely agree (1–5)
(7) Topical shifts	My thoughts were jumping from topic to topic.	Completely disagree – Completely agree (1–5)
(8) Meaningfulness	The content of my thoughts was important and meaningful to me.	Completely disagree – Completely agree (1–5)
(9) Current concerns	My thoughts were focused on uncompleted personal goals.	Completely disagree – Completely agree (1–5)
(10) Bizarreness	My thoughts were bizarre and unusual.	Completely disagree – Completely agree (1–5)
(11) Emotionality	My thoughts were emotional.	Completely disagree – Completely agree (1–5)
(12) Intentionality	My thoughts were…	(1) Engaged deliberately, with emotion (2) Came to mind spontaneously, out of nowhere
(13) Temporality	My thoughts were focused on the…	(1) Past (2) Present (3) Future (4) None of these

### Procedure

See Figure 1 for an overview schematic of the experiment procedure. Participants were recruited through Prolific (refer to the “Participants” section) and redirected to a Qualtrics survey. For compatibility with the Dormio Light website, they were required to use Google Chrome as their browser. Upon accessing the survey, participants provided informed consent, having been briefed about the study's duration, compensation, objectives, confidentiality measures, and the contact information for both the research team and the Institutional Review Board (IRB) at Duke University. Participants were told that they could withdraw from the study at any time, and that they could contact the research team or IRB with any concerns. Notably, no adverse events were reported during or after the study.

Participants then began the experiment by completing a demographics questionnaire. Following completion of this questionnaire, participants were randomly assigned to one of two initial conditions: hypnagogic dreaming or wakeful freely moving thought (FMT). Condition order was randomized and counterbalanced to avoid systematic differences that could be inherent in fixed orders, like practice effects. In both conditions, we taught participants how to use Dormio Light via an online video tutorial and written instructions, and randomly assigned either Fork or Tree (counterbalanced) as their cued object for that condition. Participants then went to the Dormio Light website and recorded prompts for themselves instructing them to think of a Fork/Tree and to report their thoughts. Following the recording of prompts, in the hypnagogia condition, they were instructed to lie down and fall into sleep, during which their pre-recorded prompts would guide them. They were cued four times (i.e., four trials, with a cue at the end of each trial) throughout an hour-long period to report their thoughts verbally for 60 s, during which the Dormio Light website recorded audio automatically. In the FMT condition, all instructions and procedures were the same, but participants were told to close their eyes and specifically not fall asleep, but rather simply let their mind wander while sitting up. Following each condition, participants reported their state of vigilance across the recently completed condition via a 3-item scale taken from Haar Horowitz et al. (2020) (“To what degree did you fall asleep, if you did?” with options “Fully asleep,” “Half asleep,” and “I did not fall asleep”). We excluded data from 52 participants who informed us that they had not remained completely awake during their FMT time, or participants who had not fallen asleep (either halfway or fully) across the hypnagogia condition. The need for these strict criteria was motivated by the fact that these states can freely flow into one another, as discussed in Tagliazucchi and Laufs (2014), Andrillon et al. (2019), and Sodré et al. (2023).

Following completion of each condition, participants returned to the Qualtrics survey and completed a manual, free-response task reporting their thoughts during and across the four-trial condition. They also uploaded available audio files of their verbal reports that were automatically downloaded to their computers; however, unexpected issues (including technological and participant errors) precluded us from analyzing these verbal reports. Participants then completed a 13-item questionnaire adapted from Smallwood et al. (2016) and Gross et al. (2020) assessing the content and structure of their thoughts. Finally, participants completed the experiment once more, but with the alternate condition (hypnagogia/FMT) and alternate object (Fork/Tree). We report results from the typed free-responses and 13-item questionnaire data.

## Results

### Thought reports: incorporations of cues

#### Effectiveness of cueing procedure

To ensure that the prompts were effective, we first sought to confirm that participants showed a higher number of incorporations of the cued item relative to the uncued item in their typed free-response thought reports. In other words, when participants were cued to think about a fork, we wanted to determine that they showed more incorporations of a fork than of a tree (and vice versa), as would be expected if the thought prompts were successful. Two raters assessed the free-response prompts for either direct (e.g., “I dreamed of becoming a tree”) or indirect (e.g., “I imagined watering big plants”) incorporation of the cued item. Correlations between the number of incorporations scored by Rater 1 and Rater 2 were very high (*r* = 0.97, *p* < 0.001); however, inter-rater reliability was relatively low (κ = 0.17). This was due to Rater 2 scoring significantly more incorporations than Rater 1 [*t*_(244)_ = 2.08, *p* = 0.03]. Despite this, primary results were unchanged when using the data from either Rater 1, Rater 2, or the average of the two raters. For simplicity, below, we report the data from Rater 1.

We verified that participants used more incorporations of their cued item than their uncued one via a significant 3-way interaction between cue (Fork, Tree), incorporation content (Fork, Tree) and incorporation type (direct, indirect) [*F*_(1, 80)_ = 60.19, *p* < 0.001, ${\;}_{\eta_{\text{p}}^{\text{2}}}$ = 0.43]. Regardless of incorporation type, participants reported significantly more incorporations of the cued item relative to the uncued item (all *p* < 0.001, all *ds* > 0.84). Tree incorporations during the Tree session (Direct: *M* = 3.09, *SD* = 2.19; Indirect: *M* = 1.72, *SD* = 1.80) and Fork incorporations during the Fork session (Direct: *M* = 2.62, *SD* = 2.60; Indirect: *M* = 1.64, *SD* = 1.96) were all significantly greater than 0 (all *p*'s < 0.001, all *d*'s > 0.84). Equally importantly, Fork incorporations during the Tree session (Direct: *M* = 0.06, *SD* = 0.37, Indirect: *M* = 0.03, *SD* = 0.16), and Tree incorporations during the Fork session (Direct: *M* = 0.01, *SD* = 0.11; Indirect: *M* = 0.06, *SD* = 0.37), were all *not* significantly different from zero (all *p*'s > 0.094, all *d*'s < 0.19). This analysis shows that the primary manipulation via the online incubation device was successful in terms of enabling participants to incorporate a particular item into their ongoing thoughts.

#### Rates of incorporation by mental state

We next asked whether the number of incorporations of the cued item differed by mental state (i.e., between hypnagogia and FMT). A 2 (condition: hypnagogia, FMT) × 2 (incorporation type: direct, indirect) repeated-measures ANOVA revealed a significant main effect of incorporation type [*F*_(1, 80)_ = 59.99, *p* < 0.001, ${\;}_{\eta_{\text{p}}^{\text{2}}}$ = 0.42], with more direct incorporations (*M* = 2.85, *SD* = 2.17) being reported than indirect incorporations (*M* = 1.68, *SD* = 1.71). There was no main effect of condition [*F*_(1, 80)_ = 0.61, *p* = 0.44, ${\;}_{\eta_{\text{p}}^{\text{2}}}$ = 0.008], suggesting that the overall number of incorporations of the cued item did not differ depending on whether cueing occurred during hypnagogia (*M* = 2.20, *SD* = 1.93) or FMT (*M* = 2.33, *SD* = 2.05). There was also no significant interaction between condition and incorporation type [*F*_(1, 80)_ = 0.11, *p* = 0.75, ${\;}_{\eta_{\text{p}}^{\text{2}}}$ = 0.001].

#### Comparing rates of incorporation between online and laboratory studies

Of the 80 participants included in the analysis, 73 (91%) reported at least one incorporation during hypnagogia, which is significantly greater than would be expected by chance [χ^2^ (1) = 54, *p* < 0.001]. This aligns well with recent in-person laboratory work: Haar Horowitz et al. (2020) found that 11 out of 12 (92%) individuals in a TDI condition reported at least one incorporation during hypnagogia, while Experiment 3 of Haar Horowitz (2022) similarly reported 23 of 25 (92%) individuals with a direct cue incorporation during hypnagogic TDI. Combined, 34 of 37 (92%) individuals from these in-person studies reported at least one incorporation of a cued item during N1 dreaming [χ^2^ (1) = 26, *p* < 0.001; Haar Horowitz et al., 2020; Haar Horowitz, 2022]. A statistical comparison of the incorporation rate between our online sample and the combined in-person samples revealed no significant difference [χ^2^ (1) = 0.01, *p* = 0.91]. As such, these results suggest that cueing during hypnagogia in an online setting yields incorporation rates comparable to those of more controlled laboratory studies, though frequentist statistics precludes us from claiming these are significantly identical.

#### Correlations between number of incorporations and dimensions of thought

We performed exploratory correlations between the number of incorporations during either condition and phenomenological dimensions of thought from that condition (see Table 1 for phenomenology questionnaire). Within hypnagogia, we observed a significant positive correlation between the number of direct incorporations and bizarreness of thought (*r* = 0.33, uncorrected *p* = 0.003). A similar result was obtained during the FMT condition (*r* = 0.26, uncorrected *p* = 0.020). All other correlations were non-significant.

### Prompt evidence and temporal coherence signatures in hypnagogia and FMT

#### Direct incorporations are related to prompt centrality

**Figure 2:**
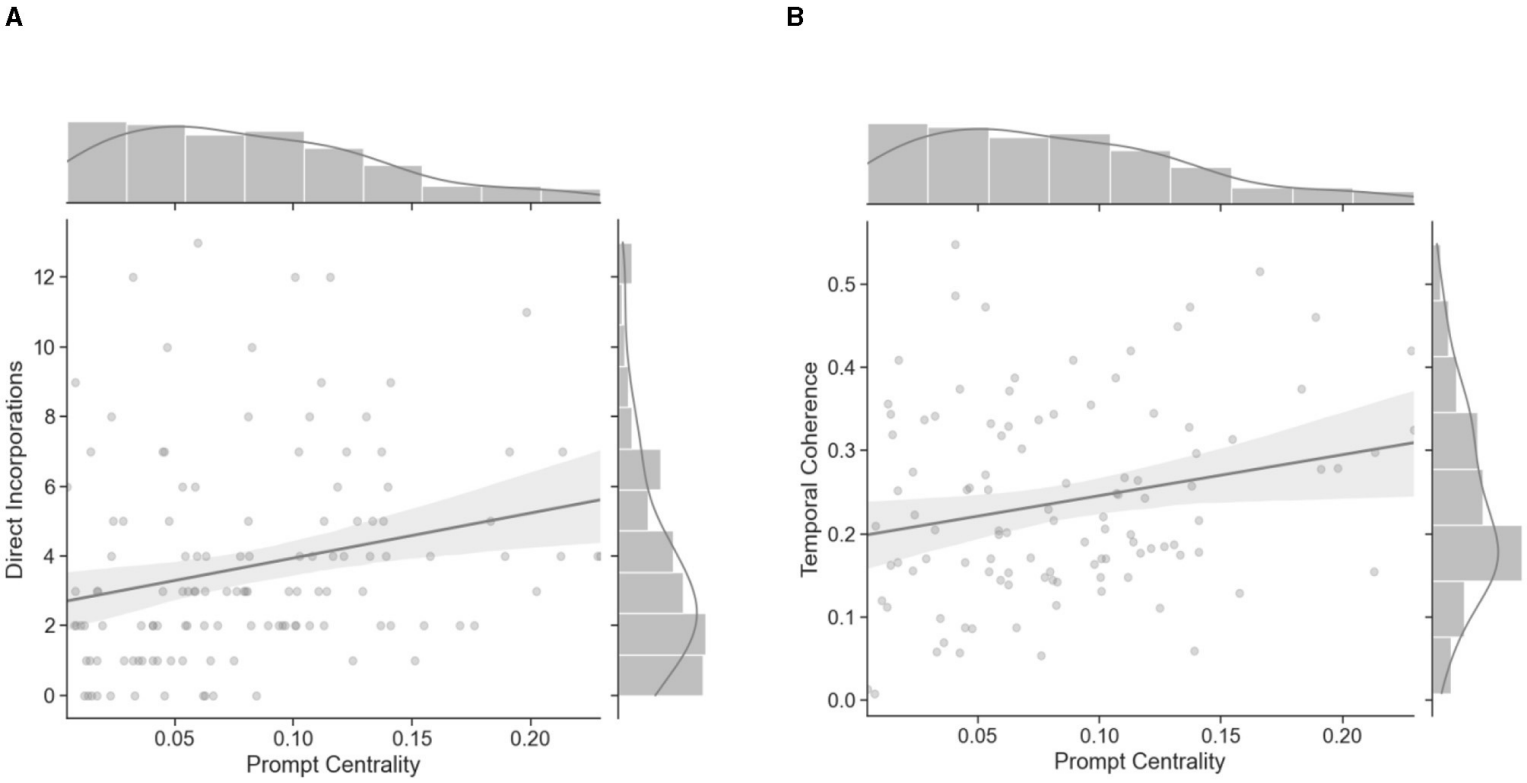
Prompt centrality is related to incorporations and linguistic structure of FMT and hypnagogic dream reports. **(A)** Prompt centrality relates to total counts of direct incorporation of the prompt across all trials across both FMT and hypnagogia conditions; **(B)** Prompt centrality is also related to temporal coherence across FMT and hypnagogia. Error bands indicate 95% CI. Marginal bars (top, right of each graph) indicate frequency distributions of each variable.

We next examined whether the centrality of the prompt—i.e., the average semantic similarity of each sentence of a report to the prompt word—would further provide us with validation of prompt incorporation in both hypnagogia and FMT. Indeed, a linear mixed model found that prompt centrality was positively related to direct incorporations [β = 0.20, standard error (SE) = 0.075, χ2 (1) = 7.1, *p* < 0.008; Figure 2A]. This finding corroborates results of the effectiveness of the cueing procedure from human ratings and suggests that prompt centrality derived from USE embeddings may reliably track content in reports across conditions/states of consciousness.

#### Prompt centrality relates to temporal coherence in FMT and hypnagogia

Both the number of incorporations of the prompt as well as prompt centrality indicate that specific content can be induced in FMT and hypnagogia, furthering validating Dormio Light's ability to implement TDI. We next investigated how incorporation of the prompt into the thought content might relate to the temporal unfolding, or structure, of FMT vs. hypnagogic dreaming. To examine this, we ran a series of linear mixed models relating prompt centrality and condition to temporal coherence. A linear mixed model found that prompt centrality was positively related to temporal coherence [β = 0.38, standard error (SE) = 0.16, χ2 (1) = 5.64, *p* = 0.018; Figure 2B], whereby greater semantic relevance to the cued prompt was related to more temporal coherence throughout the FMT and hypnagogia reports. Further, a separate linear mixed model revealed a marginal effect of condition [β = 0.02, standard error (SE) = 0.009, χ2 (1) = 3.8, *p* = 0.051], where temporal coherence was nominally higher, but not significantly so, in FMT than hypnagogia. Finally, a linear mixed model with a prompt centrality-by-condition term revealed no significant interaction [β = −0.17, standard error (SE) = 0.16, χ2 (3) = 1.2, *p* = 0.29]. These findings suggest that the incorporation of the prompt is related to greater semantic stability over time—i.e., higher sentence-by-sentence semantic similarity—of content generated during both FMT and hypnagogia. In other words, the degree to which incorporation of the cued object related to temporal coherence did not differ between conditions.

### Thought probes

#### Thought content of FMT vs. hypnagogia

We next sought to evaluate phenomenological differences between FMT and hypnagogic dreams. As such, we conducted 11 paired *t*-tests corresponding to the first 11 items in Table 1. Due to the exploratory nature of the study, we implemented two-sided, rather than one-sided, paired *t*-tests. Because of the different structures (i.e., non-continuous) of items 12 and 13, we analyzed responses for these using a binomial logistic regression and a multinomial logistic regression, respectively. To correct for multiple comparisons, while also controlling for the false discovery rate (FDR), we used the Benjamini-Hochberg corrections method (Benjamini and Hochberg, 1995). Results for the hypnagogia vs. FMT responses for the 13-item inventory are shown in Table 2. Before corrections, several variables were found to be significantly different between FMT and hypnagogia: hypnagogia was more freely moving than FMT, FMT was more intentional than dreaming, and FMT was more present-focused than hypnagogia (Intentionality and Temporality *p*-values reflect goodness-of-fit of the respective experimental models vs. baseline models). Specifically, within Temporality, participants shifting from hypnagogia to FMT were 87.6% more likely to have their thoughts remain in the present compared to a baseline atemporal state (*p* = 0.011). After multiple comparison corrections for items 1–13, no significance survives.

**Table 2:** Hypnagogic dreaming vs. FMT item-level contrasts.

**Item**	**Hypnagogia – FMT estimate**	**Raw two-sided *p*-value**	**BH-adjusted *p*-value**
Freedom of thought flow	0.3125	**0.018**	0.137
Intentionality^*^	N/A	**0.027**	0.137
Temporality^*^	N/A	**0.032**	0.137
Words	−0.2375	0.138	0.445
Bizarreness	0.1750	0.171	0.445
Current concerns	−0.1625	0.334	0.652
Novelty	0.1250	0.430	0.652
Emotionality	−0.1250	0.438	0.652
Negative valence	−0.1000	0.465	0.652
Images	0.0625	0.518	0.652
Meaningfulness	−0.1000	0.552	0.652
Topical shifts	0.0500	0.726	0.753
Positive valence	0.0375	0.753	0.753

^*^p-values come from logistic regression goodness-of-fit tests, not paired *t*-tests. Bold signifies *p*-value below 0.05. FMT = Freely moving thought. BH = Benjamini-Hochberg.

#### Partial correlations

We ran partial Pearson's *r* correlations on items 1–11 of the thought probe battery for each condition, in addition to participant age and number of hypnagogic dreams reported during the hypnagogia task (see Supplementary Table A for full matrix). Partial correlations were assessed due to the possibility that several other facets of thought influence one another in confounding ways; hence, in a partial correlation we control for every other variable besides the two of interest. Items 12 and 13 of the thought probe battery were not included due to their non-continuous structure.

**Figure 3:**
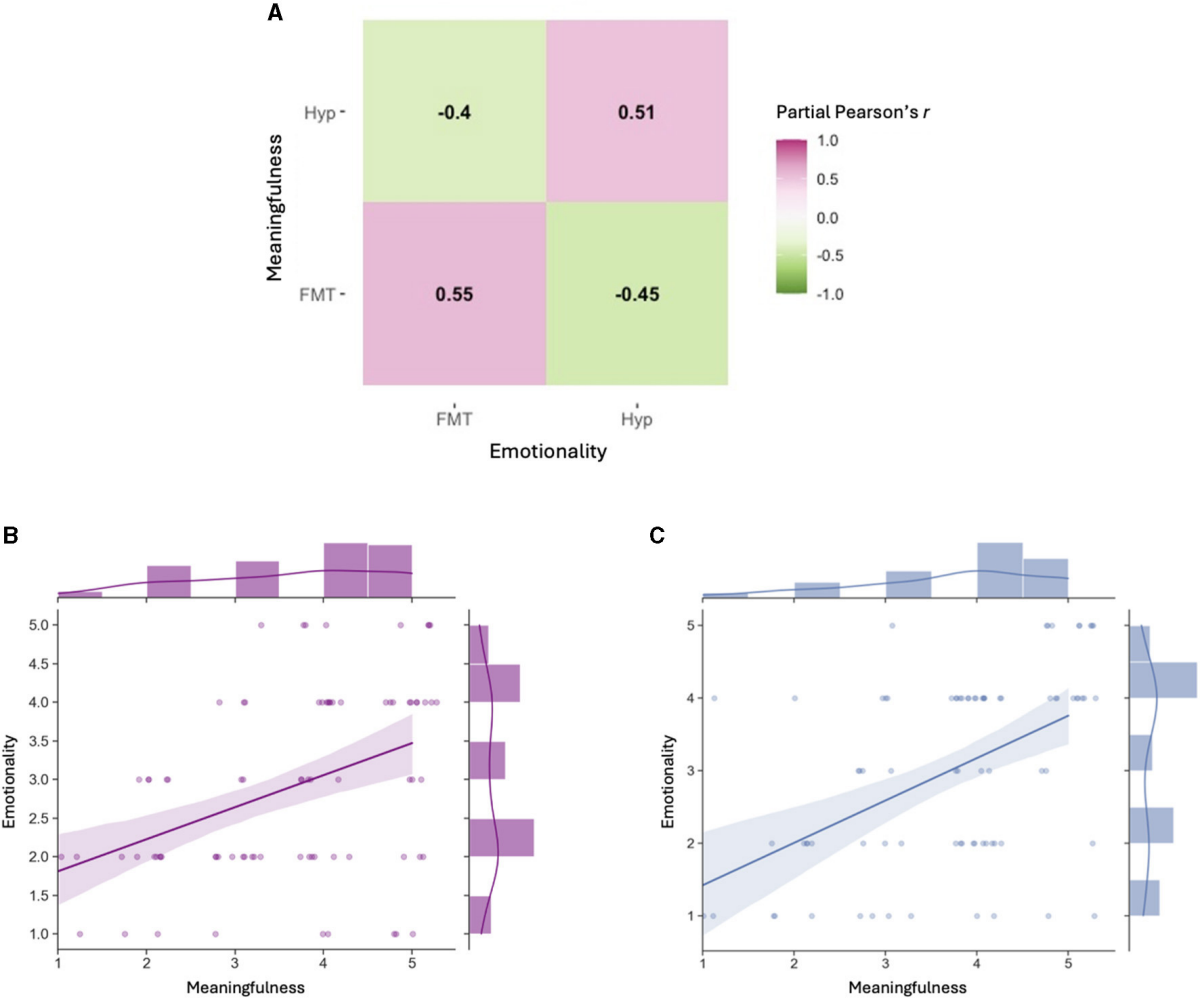
Emotionality and Meaningfulness partial correlations between- and within- conditions. **(A)** Heatmap displaying Emotionality and Meaningfulness are positively correlated with one another within each condition (e.g., Hypnagogia [Hyp] Emotionality positively associated with Hypnagogia Meaningfulness), but negatively correlated across conditions (e.g., Hypnagogia Emotionality negatively associated with FMT Meaningfulness). **(B)** Scatter plot of ratings for Hypnagogia Meaningfulness against Hypnagogia Emotionality (*r* = 0.51, *p* < 0.001). **(C)** Scatter plot of ratings for FMT Meaningfulness against FMT Emotionality (*r* = 0.55, *p* < 0.001). Error bands indicate 95% CI. Marginal bars (top, right of each graph) indicate frequency distributions of each variable.

Many partial correlations emerged as significant, and we discuss a subset below. Of note is that higher Emotionality of thoughts was associated with higher Meaningfulness of thoughts *within* each condition (Meaningful_Hyp_~Emotional_Hyp_: *r* = 0.51, *p* < 0.001; Meaningful_FMT_~Emotional_FMT_: *r* = 0.55, *p* < 0.001). However, *between* conditions, correlations were reversed, where higher Emotionality correlated with lower scores of Meaningfulness (Meaningful_Hyp_~Emotional_FMT_: *r* = −0.40, *p* = 0.002; Meaningful_FMT_~Emotional_Hyp_: *r* = −0.45, *p* < 0.001). This suggests differences between states of hypnagogia and FMT in how they associate emotion and meaning with one another (Figure 3). Additionally, the Bizarreness of hypnagogic dreams significantly (and positively) correlated with the Novelty of hypnagogic dreams (*r* = 0.43, *p* = 0.005), but such a relationship was not seen within FMT (*r* = 0.22, *p* = 0.10). Number of dreams reported was also significantly correlated with the Novelty of hypnagogic dreams (*r* = 0.31, *p* = 0.019). We also found that as age increased, participants tended to experience more-vivid hypnagogic dreams (*r* = 0.28, *p* = 0.035), but less-vivid FMT thoughts (*r* = −0.30, *p* = 0.023).

#### Exploratory factor analysis

We input all continuous variables (items 1–11) into an Exploratory Factor Analysis (EFA), for each condition, to assess potential underlying factors onto which variables may jointly load.^2^ The EFA technique is often used in psychometrics and questionnaire design in determining sub-scales, but can be utilized in designs with sufficient and appropriately structured data wishing to explore latent groupings of variables (Thompson and Daniel, 1996). Accordingly, this EFA procedure was done for two primary reasons: first, we wished to further measure phenomenological differences between our two examined conditions using an exploratory but robust model-fitting statistical technique; second, we wanted to develop new and fewer data-driven items (i.e., factors) that could replace our exploratory items from Table 1 in future research. Such factor analyses are frequently done in mind-wandering literatures for these reasons (e.g., Ruby et al., 2013; Smallwood et al., 2016). We ran two separate EFAs, one for each condition, due to the repeated-measures design of the study. Thus, with two EFAs, different loadings in each condition reflect phenomenologically different latent structures of responses (items 1–11) in each cognitive state.

To initially determine the number of factors for each condition, we ran a parallel analysis.^3^ Using a common-factors model in each parallel analysis, we found that data from the hypnagogia condition could best be loaded onto 4 factors, and the FMT data onto 5 factors (see Supplementary Figures 1, 2). We then ran an EFA on the data from each condition specifying these respective number of factors, using maximum likelihood as the factor method and oblimin as the rotation method. Oblimin is a common type of an oblique rotation method, which allows factors to be freely correlated instead of forced into orthogonalization; this generally allows for greater fit of the data and satisfies the exploratory aim of this study (Henson and Roberts, 2006). Results of standardized loadings and variance for each condition are shown in Tables 3a, b, 4a, b. In line with the literature on EFA loadings (see Henson and Roberts, 2006), we considered coefficients with magnitudes equal to or greater than 0.40 as substantial. Factor loadings in each EFA were good and clearly delineated latent groupings among different items; only one cross-factor loading (item with a substantial loading in more than one factor) emerged in the FMT EFA.

**Table 3A:** Standardized EFA loadings, hypnagogia.

**Variable**	**Factor 1 (Valence)**	**Factor 2 (Unusualness)**	**Factor 3 (Explicit concerns)**	**Factor 4 (Emotional intensity)**	***h* ^2^**
Positive valence	**−0.75**	−0.05	0.00	0.15	0.60
Negative valence	**0.93**	0.00	−0.03	0.09	0.87
Structure: images	0.22	−0.06	−0.26	0.11	0.12
Structure: words	−0.06	0.02	**0.84**	0.00	0.71
Novelty	−0.10	**0.79**	0.03	0.06	0.61
Freedom of thought flow	0.05	0.24	0.08	0.14	0.09
Meaningfulness	−0.17	−0.09	0.14	**0.46**	0.29
Topical shifts	0.13	0.30	0.17	−0.09	0.14
Current concerns	**0.41**	−0.10	**0.48**	0.05	0.42
Bizarreness	0.13	**0.73**	−0.03	−0.05	0.58
Emotionality	0.04	0.03	−0.02	**0.81**	0.66

Loading coefficients greater than absolute value of 0.40 are bolded as indicators of substantial loadings. *h*^2^ refers to the communality coefficient for a respective variable, i.e., its contribution towards the overall variance.

**Table 3B:** EFA loadings variance descriptions, hypnagogia.

	**Factor 1 (Valence)**	**Factor 2 (Unusualness)**	**Factor 3 (Explicit concerns)**	**Factor 4 (Emotional intensity)**
Sum of squares loadings	1.74	1.32	1.06	0.96
Proportion of variance	0.16	0.12	0.10	0.09
Cumulative variance	0.16	0.28	0.37	0.46
Proportion explained	0.34	0.26	0.21	0.19
Cumulative proportion	0.24	0.60	0.81	1.00

**Table 4A:** Standardized EFA loadings, FMT.

**Variable**	**Factor 1 (Valence)**	**Factor 2 (Emotional intensity)**	**Factor 3 (Unusualness)**	**Factor 4 (Concerns)**	**Factor 5 (Form of thought)**	***h* ^2^**
Positive valence	**−0.64**	0.22	−0.05	−0.14	−0.04	0.53
Negative valence	**1.00**	0.05	0.02	−0.03	−0.03	1.00
Structure: images	−0.13	0.02	0.11	−0.02	**0.51**	0.29
Structure: words	0.00	**0.44**	0.07	0.29	**−0.42**	0.43
Novelty	−0.15	0.09	0.36	−0.05	0.16	0.16
Freedom of thought flow	−0.09	0.23	0.04	0.27	**0.46**	0.35
Meaningfulness	−0.07	**0.81**	−0.02	−0.06	−0.01	0.66
Topical shifts	0.01	−0.09	0.32	**0.43**	−0.05	0.34
Current concerns	0.04	−0.05	−0.08	**0.71**	0.03	0.50
Bizarreness	0.02	−0.01	**0.99**	−0.02	0.00	1.00
Emotionality	0.22	**0.55**	−0.02	**0.40**	**0.60**	0.60

Loading coefficients greater than absolute value of 0.40 are bolded as indicators of substantial loadings. *h*^2^ refers to the communality coefficient for a respective variable, i.e., its contribution toward the overall variance.

**Table 4B:** EFA loadings variance descriptions, FMT.

	**Factor 1 (Valence)**	**Factor 2 (Emotional intensity)**	**Factor 3 (Unusualness)**	**Factor 4 (Concerns)**	**Factor 5 (Form of thought)**
Sum of squares loadings	1.51	1.30	1.26	0.90	0.87
Proportion of variance	0.14	0.12	0.11	0.08	0.08
Cumulative variance	0.164	0.26	0.37	0.45	0.53
Proportion explained	0.26	0.22	0.22	0.15	0.15
Cumulative proportion	0.26	0.48	0.70	0.85	1.00

Across the results of each EFA, some similarities emerged that reflected shared properties of the data across conditions. For instance, Factor 1 (Valence) across both conditions highly loaded Positivity and Negativity, Factor 2_Hyp_/Factor 3_FMT_ (Unusualness) consisted of Bizarreness, and Factor 4_Hyp_/Factor 2_FMT_ (Emotional Intensity) reflected a latent variable shared primarily between Emotionality and Meaningfulness.

However, the factor analyses also supported differences between each condition, as seen in the different factors produced and specific item loadings within these factors. For instance, the FMT EFA produced a factor seemingly key in the structure of thoughts (Factor 5_FMT_: high loadings in Images, Words, Freedom of thought, and Emotionality), while no such factor is evident in the hypnagogia EFA, suggesting reduced modally organized structure in the latter state. In addition, though both EFAs produced factors related to Unusualness (Factor 2_Hyp_/Factor 3_FMT_), the hypnagogia factor involved loadings heavy in both Novelty and Bizarreness, while the FMT factor only consisted of Bizarreness. As such, in our sample, Novelty and Bizarreness were more associated with one another in hypnagogia than FMT (this is also supported by earlier partial correlation results, see Supplementary Table A).

## Discussion

In the present study, we developed a novel, relatively hardware-free tool that appears to be effective at cueing and incubating specific content in hypnagogic dreaming and FMT. This was motivated by our desire to expand the accessibility and portability of both sleep research (which is burdened by expensive equipment and in-person procedures; e.g., Escourrou et al., 2000) and FMT research. Secondly, we examined the phenomenological differences between hypnagogic and FMT thought, as measured by thought probes and *post-hoc* free-response reports using both human rating and semantic modeling (USE) methods.

Our findings reflected successful cueing and incubation of thought content across both wake (FMT) and sleep (hypnagogia) conditions. Participants appropriately incorporated cued items as determined by both hand-rating and semantic modeling (USE) analyses. Further, our overall incorporation rate using remote TDI was relatively high: 73 of 80 (91%) of our participants reported at least one incorporation of the cued item. These results are comparable to in-person TDI results, where 34 of 37 (92%) reported at least one incorporation of the cued item (Haar Horowitz et al., 2020; Haar Horowitz, 2022). These proportions across studies were non-significantly different, underscoring the success of the remote procedure that further collected over double the sample size. In this way, our hypotheses regarding the feasibility of Dormio Light online dream research were supported, potentially assuaging resource-related risks of sleep-research listed in Burgdorf et al. (2018), Escourrou et al. (2000), and Topalidis et al. (2023). Dormio Light can expand incubation research in a manner that eases time and financial burdens, while also opening new windows of opportunity for an examination of FMT behavior in multiple ways. Firstly, with cued prompts, we can now control for the “ignition point” (or onset of) FMT and disentangle it from the continuation of thought, a point raised by Smallwood (2013) that highlights the inherent difficulty in studying dynamics of mind-wandering. Secondly, to our knowledge, Dormio Light is the first tool to permit experimental control over the thought content of FMT, and future work can investigate the relationship between FMT and other theoretically interesting variables like wellbeing and creativity (see “Future work” below).

Additionally, we compared phenomenological profiles of hypnagogic and FMT content. If FMT and hypnagogic dreaming states rely on a continuous psychological process that gradually shifts across states (i.e., the “continuity hypothesis of dreaming”; Schredl and Hofmann, 2003), then intermediate states (i.e., those temporally adjacent to one another like FMT and hypnagogia) should not greatly differ from one another (Sodré et al., 2023); yet, these states should also be separable to a certain extent. Our results suggest such separability, though only before correcting for multiple comparisons: hypnagogic dreaming was less intentional (also corroborating views that FMT is more constrained than dreaming; Christoff et al., 2016), more freely flowing, and less present-focused than FMT. We specifically supported past work (Fox et al., 2013; van Rijn et al., 2015; Smallwood et al., 2016) that has found that forms of mind-wandering revolve around current and relevant concerns, while dreaming consolidates non-present (i.e., past) information. However, as mentioned, our claims are limited given the exploratory nature of our paradigm that demanded strict corrections of potentially inflated *p*-values. Thus, in future research, a pre-registered study with directional hypotheses may assuage such concerns. In addition, qualitative differences in EFA latent structures hint at differential structure of thoughts in each state, as well as distinct associations of Bizarreness and Novelty.^4^ The relationships between Emotionality and Meaningfulness also showed a positive association between the two variables within conditions, but between conditions this association was negative. Overall, this suggests that we targeted distinct states via the Dormio Light website, as captured by subtle differences that–while aligning with past work and mirroring the subtle experiential differences and temporal nearness of the states—should be further understood in future work.

Equally interesting, hypnagogia and FMT were not significantly different across several properties. For 10 of the 13 items of our phenomenology battery, no significant differences across hypnagogia and FMT emerged before corrections for multiple comparisons. Included in these null comparison effects were features of valence and emotion. While past work has shown that hypnagogia is generally less emotional than REM dreaming (Ghibellini and Meier, 2023), it is possible that hypnagogia is similar in emotionality as FMT given their proximity to one another.

In sum, we found some instances of phenomenological differences in mentation states, perhaps reflecting the fact that hypnagogia is a neighbor both temporally and functionally to FMT. These states of consciousness may flow into and build upon one another in certain features of thought that could be explored in future experiments. However, it must be noted that we did not assess more than two states of consciousness and therefore cannot fully explore how phenomenological and structural characteristics of thought content shift as multiple states progress (as in Gross et al., 2020); thus, we cannot confidently confirm or deny a comprehensive continuity hypothesis of dreaming.

### Limitations and considerations

Our study is subject to some notable limitations that should be corrected in future work. Firstly, the lack of physiological sensing in our method means we must trust participants' subjective report of sleep vs. wake. There is no objective (non-self-report) measure of sleep stage possible with our remote Dormio Light methodology, thus precluding Dormio Light from targeting hypnagogia as traditionally defined via polysomnography or via the original Dormio glove. Dormio Light is a tool for researchers who consider the tradeoff for ecological validity and ease of use to be worth the lack of physiological or neural sleep staging. To maximize our confidence that participants were truly in sleep vs. waking states without physiological data, we relied on strict self-report criteria for analysis inclusion in an effort to provide as conservative of findings as possible. This method eliminated data from 52 participants, which may indicate that, although our method is much easier to use for hypnagogic research in terms of resources and convenience, the timer method is likely less sensitive to hypnagogic signals than the Dormio glove/muscle-flexion method or polysomnography (hence, there is a notable tradeoff that researchers ought to consider).

A second limitation is that assessing and rating incorporation in dreams is always a task of some difficulty. As is applicable to all dream research, we must consider the validity of participants' *post-hoc* reports, as “dreams can be forgotten or fabricated due to demand characteristics” (Haar Horowitz et al., 2020, p. 10). However, optimistically, some studies have found hypnagogic content to be well-retained and remembered when participants are awoken (Hori et al., 1994). Additionally, when rating these reports, it is important to bear in mind that a cue can enter a dream as an association (e.g., a cued tree might become an image of branching dendrites), which might not be evident to raters and, thus, might fail to be identified as an incorporation. We are encouraged that our computational measure of prompt centrality was positively related to human ratings of direct incorporations (*p* < 0.008) across both conditions, but future research should explore limitations on measuring incorporation.

Third, there is still debate as to how cued mind-wandering (an umbrella concept of our cued FMT) differs in flow and structure compared to other, more-spontaneous forms of mind-wandering (see Seli et al., 2016 for a review); future studies may investigate this distinction specifically and compare hypnagogic thought with different modes of mind-wandering.

Fourth, our Dormio Light website was specifically made for the Chrome browser at the time of the study, and some participants indicated difficulty uploading audio recordings. For scaled-up research across labs, further investment in compatibility and interoperability will be necessary.

On a final note, we did not pre-screen participants for psychiatric or sleep disorders. Importantly, however, participants who are predisposed to psychotic disorders may be at increased risk of psychotic decompensation when engaging in hypnagogia or mind-wandering.^5^ Fortunately, in our study, no participants reported any such adverse events. In any case, to maximize participant safety, future research utilizing Dormio Light would do well to screen for any history of psychotic disorders (e.g., schizophrenia, schizoaffective disorder) or sleep disorders and related symptoms (e.g., narcolepsy, isolated hypnagogic/hypnopompic hallucinations, recurrent sleep paralysis, parasomnias).

### Future work

Given the relatively successful implementation of Dormio Light, future research can investigate the experience of, and mechanisms behind, hypnagogia more in-depth. Moreover, the same could be done for research on FMT, which, before this study, lacked a tool with which to effectively cue and capture such thought content. This novel resource, given its experimental ability to control thought content and ignition, can help open a window into the differential (or similar) mechanisms that underly mnemonic and creative effects of these forms of spontaneous thinking.

Though the early mind-wandering literature focused on detrimental effects of inattention on cognitive performance and mood (Killingsworth and Gilbert, 2010; Reichle et al., 2010), the newer literature has since emphasized the possible benefits of inattentive mind-wandering including creativity, future-planning, and memory consolidation (Baird et al., 2012; Seli et al., 2018; Dobson and Christoff, 2020). Hypnagogia (and dreaming in general) also shows effects of memory and learning consolidation (Wamsley, 2014; Antrobus and Wamsley, 2017; Barrett, 2017), including across linguistic tasks (De Koninck et al., 1990) and motor coordination tasks (Wamsley et al., 2010; Fogel et al., 2018; Wamsley and Stickgold, 2019). To understand the differential roles of memory systems across forms of spontaneous thoughts like FMT and hypnagogia, Dormio Light lends itself well to memory experiments. For instance, research could present to-be-remembered words during both hypnagogia and FMT as permitted in the audio recordings of the website, and subsequently compare later recollection.

The fascinating role of memory and learning in spontaneous thought like mind-wandering and hypnagogia further extend to cognitive processes of creativity and creative problem-solving. Mind-wandering has been investigated as a source of creative incubation and inspiration. For instance, Irving et al. (2022) showed boosted creativity during FMT, the form of mind-wandering investigated in this study, by showing participants a moderately engaging movie clip, during which creative incubation was predicted to occur. Thus, future research could implement Dormio Light to specifically investigate how cue content influences FMT dynamics, thought constraint, and resultant creativity and problem-solving. Meanwhile, hypnagogia has also been shown to afford unique insight in creative problem-solving tasks. In a recent study on mathematical processing during hypnagogia by Lacaux et al. (2021), participants were given equations to solve, but the problems actually had a hidden rule that would immediately provide the answer. Remarkably, 83% of participants who spent at least 15 s in hypnagogia discovered the hidden rule, compared to 30% of those in wakeful mind-wandering and 14% in N2. With Dormio Light, similar research may be carried out remotely across other creative and problem-solving domains at a larger scale.

Our use of multiple statistical techniques that converged on general findings also provides possibilities for forays into both additional self-report questionnaires and more automated assessment of dream and FMT content. Regarding the former, given the exploratory thought-probe battery utilized here (though initially adapted from Smallwood et al., 2016 and Gross et al., 2020), our EFA results point toward a more data-driven and targeted comparison of states with fewer items. Future work would thus do well to investigate the latent factors suggested by our data and the factor analyses. Regarding the latter, given the success of the USE text-embedding model, a promising direction for future research is to test differences in the semantic structure across dreaming and other types of mental content. Contemporary language embedding models, such as USE, may be further leveraged to derive differences in dream content for more selective semantic representations, such as similarity to peripheral features (e.g., food rather than fork; the smell of a forest rather than a tree). Furthermore, future research could probe other linguistic relationships, such as causal language, which may be more abstractly represented in dream content. Given that recent research has shown that trait creativity is related to an ability to generate semantically divergent linguistic content (Beaty and Johnson, 2021; Olson et al., 2021), future work might also explore how measures of semantic stability in dream content, such as temporal coherence, might also be related to trait creativity.

## Concluding remarks

Our study reports successful use of a novel tool (Dormio Light) for targeted incubation of cued themes across both hypnagogia and FMT. Dormio Light lends itself to collection from large pools of participants across the world—that are accessible much faster than studies using in-person cueing—for similar studies. We are particularly excited that this method mirrored in-person patterns of results in terms of incubation rates. We further discovered potential differences in features of thought between the FMT and hypnagogia conditions, including intentionality, freely flowing nature, and temporality, but only to a limited extent that did not survive corrections for multiple comparisons. Still, significant differences appeared in correlational data, suggesting different latent structures of thought and perhaps functional differences between the two states. This contributes to empirical investigations of the continuity hypothesis of dreaming, and affords a more in-depth exploration into mentation features and underlying processes across states of consciousness. Moving forward, we believe Dormio Light will serve as a useful tool in research on hypnagogia and mind-wandering, allowing for new and exciting investigations into the mechanisms underlying these altered states of consciousness, and permitting the development of thought-cueing methods that can be used to allow people to harness the power of their untethered minds across wake and sleep.

## Data availability statement

The raw data supporting the conclusions of this article will be made available by the authors, without undue reservation.

## Ethics statement

The studies involving humans were approved by the Duke University Campus Institutional Review Board. The studies were conducted in accordance with the local legislation and institutional requirements. The participants provided their written informed consent to participate in this study.

## Author contributions

LB: Conceptualization, Data curation, Formal analysis, Investigation, Methodology, Project administration, Validation, Visualization, Writing – original draft, Writing – review & editing. AH: Conceptualization, Investigation, Methodology, Project administration, Resources, Software, Writing – original draft, Writing – review & editing. MM: Data curation, Formal analysis, Methodology, Visualization, Writing – review & editing, Writing – original draft. RB: Writing – review & editing. DD: Data curation, Formal analysis, Validation, Writing – review & editing. CC: Conceptualization, Software, Writing – review & editing. PM: Conceptualization, Project administration, Resources, Supervision, Writing – review & editing. PS: Writing – original draft, Writing – review & editing, Conceptualization, Funding acquisition, Investigation, Methodology, Project administration, Resources, Supervision.

## Footnotes

^1^Some participants used the free-response prompt to also report things unrelated to the content of their thoughts in the condition, including questions about the study, Prolific-specific procedures, bugs they encountered, etc. These mentions were cleaned out.

^2^Note that the EFA procedure demands continuous variables due to the initial computation of assessing shared variance via correlation; hence the exclusion of items 12 and 13.

^3^Parallel analyses create simulated data based on the structure and range of the actual data (Horn, 1965). Eigenvalues from this simulated data are compared to those of the actual data and are compared across the range of possible factors. We then retain the number of factors where observed eigenvalues are greater than the simulated eigenvalues, as typically visualized by a scree plot (Sakaluk and Short, 2017); this step provides the number of factors that the observed data best discretize onto. See Supplementary Figures 1, 2.

^4^These factor analyses–which mirror work by Ruby et al. (2013) and Smallwood et al. (2016) in reference to the FMT loadings of Affect and of Thought Structure, respectively– can motivate new research by inspiring new scales to test such latent factors (as recommended by Ghibellini and Meier, 2023).

^5^We thank a reviewer for this comment.

## References

[B1] Andrillon T. Windt J. Silk T. Drummond S. P. A. Bellgrove M. A. Tsuchiya N. (2019). Does the mind wander when the brain takes a break? Local sleep in wakefulness, attentional lapses and mind-wandering. Front. Neurosci. 13:949. 10.3389/fnins.2019.00949 31572112 PMC6753166

[B2] Antrobus J. S. Wamsley E. J. (2017). Sleep mentation in REM and NREM: a cognitive neuroscience perspective. Ref. Mod. Neurosci. Biobehav. Psychol. 10.1016/B978-0-12-809324-5.02859-5

[B3] Baird B. Smallwood J. Mrazek M. D. Kam J. W. Y. Franklin M. S. Schooler J. W. (2012). Inspired by distraction: mind wandering facilitates creative incubation. Psychol. Sci. 23, 1117–1122. 10.1177/0956797612446024 22941876

[B4] Barrett D. (2017). Dreams and creative problem-solving. Annal. N. Y. Acad. Sci. 1406, 64–67. 10.1111/nyas.13412 28640937

[B5] Beaty R. E. Johnson D. R. (2021). Automating creativity assessment with SemDis: an open platform for computing semantic distance. Behav. Res. Methods 53, 757–780. 10.3758/s13428-020-01453-w 32869137 PMC8062332

[B6] Benjamini Y. Hochberg Y. (1995). Controlling the false discovery rate: a practical and powerful approach to multiple testing. J. R. Stat. Soc. Ser. B Methodol. 57, 289–300. 10.1111/j.2517-6161.1995.tb02031.x

[B7] Burgdorf A. Güthe I. Jovanović M. Kutafina E. Kohlschein C. Bitsch J. Á.. (2018). The mobile sleep lab app: an open-source framework for mobile sleep assessment based on consumer-grade wearable devices. Comp. Biol. Med. 103, 8–16. 10.1016/j.compbiomed.2018.09.025 30316065

[B8] Carr M. Solomonova E. (2019). “Dream recall and content in different sleep stages and time-of-night effect,” in Dreams: Biology, Psychology and Culture, eds. K. Valli, R. Hoss, and R. Gongloff (Santa Barbara, CA: Greenwood Publishing Group), 167–172.

[B9] Carskadon M. A. Dement W. C. (1982). The multiple sleep latency test: What does it measure? Sleep: J. Sleep Res. Med. 5, 67–72. 10.1093/sleep/5.S2.S67 7156656

[B10] Castaldo V. Holzman P. S. (1967). The effects of hearing one's own voice on sleep mentation. The J. Nerv. Mental Dis. 144, 2–13. 10.1097/00005053-196701000-00002 5337101

[B11] Cer D. Yang Y. Kong S. Hua N. Limtiaco N. John R. S.. (2018). “Universal sentence encoder for English,” in Proceedings of the 2018 Conference on Empirical Methods in Natural Language Processing: System Demonstrations (Brussels: Association for Computational Linguistics), 169–174. 10.18653/v1/D18-2029

[B12] Christoff K. Irving Z. C. Fox K. C. R. Spreng R. N. Andrews-Hanna J. R. (2016). Mind-wandering as spontaneous thought: a dynamic framework. Nat. Rev. Neurosci. 17, 718–731. 10.1038/nrn.2016.113 27654862

[B13] De Koninck J. Christ G. Héber G. Rinfret N. (1990). Language learning efficiency, dreams and REM sleep. Psychiatr. J. Univ. Revue de psychiatrie de l'Universite d'Ottawa 15, 91–92. 2374794

[B14] Dobson C. Christoff K. (2020). “Productive mind wandering in design practice,” in Creativity and the Wandering Mind, eds. D. D. Preiss, D. Cosmelli, and J. C. Kaufman (New York, NY: Academic Press), 271–281.

[B15] Escourrou P. Luriau S. Rehel M. Nédelcoux H. Lanoë J.-L. (2000). Needs and costs of sleep monitoring. Stud. Health Technol. Inform. 22, 69–85. 11151608

[B16] Fogel S. M. Ray L. B. Sergeeva V. De Koninck J. Owen A. M. (2018). A novel approach to dream content analysis reveals links between learning-related dream incorporation and cognitive abilities. Front. Psychol. 9:1398. 10.3389/fpsyg.2018.01398 30127760 PMC6088287

[B17] Fox K. C. R. Nijeboer S. Solomonova E. Domhoff G. W. Christoff K. (2013). Dreaming as mind wandering: evidence from functional neuroimaging and first-person content reports. Front. Hum. Neurosci. 7:412. 10.3389/fnhum.2013.00412 23908622 PMC3726865

[B18] Ghibellini R. Meier B. (2023). The hypnagogic state: a brief update. J. Sleep Res. 32:e13719. 10.1111/jsr.13719 36017720 PMC10078162

[B19] Gross M. E. Smith A. P. Graveline Y. M. Beaty R. E. Schooler J. W. Seli P. (2020). Comparing the phenomenological qualities of stimulus-independent thought, stimulus-dependent thought and dreams using experience sampling. Philos. Trans. Royal Soc. Biol. Sci. 376:20190694. 10.1098/rstb.2019.0694 33308068 PMC7741088

[B20] Haar Horowitz A. Cunningham T. J. Maes P. Stickgold R. (2020). Dormio: a targeted dream incubation device. Conscious. Cognit. 83:102938. 10.1016/j.concog.2020.102938 32480292 PMC7590944

[B21] Haar Horowitz A. H. Esfahany K. Gálvez T. V. Maes P. Stickgold R. (2023). Targeted dream incubation at sleep onset increases post-sleep creative performance. Sci. Rep. 13:31361. 10.1038/s41598-023-31361-w 37188795 PMC10185495

[B22] Haar Horowitz A. J. (2022). Interfacing with dreams: novel technologies and protocols for targeted dream incubation (Doctoral dissertation). Cambridge, MA: Massachusetts Institute of Technology.

[B23] Henson R. K. Roberts J. K. (2006). Use of exploratory factor analysis in published research: common errors and some comment on improved practice. Educ. Psychol. Measur. 66, 393–416. 10.1177/0013164405282485

[B24] Hori T. Hayashi M. Morikawa T. (1994). Topographical EEG Changes and the Hypnagogic Experience. Sleep Onset: Normal and Abnormal Processes. New York, NY: American Psychological Association, 237–253.

[B25] Horn J. L. (1965). A rationale and test for the number of factors in factor analysis. Psychometrika 30, 179–185. 10.1007/BF02289447 14306381

[B26] Horner R. L. Sanford L. D. Pack. A. I Morrison A. R. (1997). Activation of a distinct arousal state immediately after spontaneous awakening from sleep. Brain Res. 778, 127–134. 10.1016/S0006-8993(97)01045-7 9462884

[B27] Irving Z. C. McGrath C. Flynn L. Glasser A. Mills C. (2022). The shower effect: Mind wandering facilitates creative incubation during moderately engaging activities. Psychol. Aesthetics Creativity Arts. 10.1037/aca0000516. [Epub ahead of print].

[B28] Killingsworth M. A. Gilbert D. T. (2010). A wandering mind is an unhappy mind. Science 330:932. 10.1126/science.1192439 21071660

[B29] Lacaux C. Andrillon T. Bastoul C. Idir Y. Fonteix-Galet A. Arnulf I.. (2021). Sleep onset is a creative sweet spot. Sci. Adv. 7:5866. 10.1126/sciadv.abj5866 34878849 PMC8654287

[B30] McVay J. Kane M. (2013). Dispatching the wandering mind? Toward a laboratory method for cuing “spontaneous” off-task thought. Front. Psychol. 4:570. 10.3389/fpsyg.2013.00570 24027542 PMC3760067

[B31] Mills C. Raffaelli Q. Irving Z. C. Stan D. Christoff K. (2018). Is an off-task mind a freely-moving mind? Examining the relationship between different dimensions of thought. Conscious. Cognit. 58, 20–33. 10.1016/j.concog.2017.10.003 29107470

[B32] Mota-Rolim S. A. Brandão D. S. Andrade K. C. de Queiroz C. M. T. Araujo J. F. de Araujo D. B. Ribeiro S. (2015). Neurophysiological features of lucid dreaming during N1 and N2 sleep stages: two case reports. Sleep Sci. 4:215. 10.1016/j.slsci.2016.02.093

[B33] Olson J. A. Nahas J. Chmoulevitch D. Cropper S. J. Webb M. E. (2021). Naming unrelated words predicts creativity. Proc. Nat. Acad. Sci. 118:e2022340118. 10.1073/pnas.2022340118 34140408 PMC8237676

[B34] Perogamvros L. Baird B. Seibold M. Riedner B. Boly M. Tononi G. (2017). The phenomenal contents and neural correlates of spontaneous thoughts across wakefulness, NREM sleep, and REM sleep. J. Cognit. Neurosci. 29, 1766–1777. 10.1162/jocn_a_01155 28562209 PMC6390836

[B35] Quercia A. Zappasodi F. Committeri G. Ferrara M. (2018). Local use-dependent sleep in wakefulness links performance errors to learning. Front. Hum. Neurosci. 12:122. 10.3389/fnhum.2018.00122 29666574 PMC5891895

[B36] Reichle E. D. Reineberg A. E. Schooler J. W. (2010). Eye movements during mindless reading. Psychol. Sci. 21, 1300–1310. 10.1177/0956797610378686 20679524

[B37] Ruby F. J. M. Smallwood J. Engen H. Singer T. (2013). How self-generated thought shapes mood—the relation between mind-wandering and mood depends on the socio-temporal content of thoughts. PLoS ONE 8:e77554. 10.1371/journal.pone.0077554 24194889 PMC3806791

[B38] Sakaluk J. K. Short S. D. (2017). A methodological review of exploratory factor analysis in sexuality research: used practices, best practices, and data analysis resources. J. Sex Res. 54, 19. 10.1080/00224499.2015.1137538 26886499

[B39] Schacter D. L. (1976). The hypnagogic state: A critical review of the literature. Psychol. Bullet. 83, 452–481. 10.1037/0033-2909.83.3.452 778884

[B40] Schredl M. Hofmann F. (2003). Continuity between waking activities and dream activities. Conscious. Cognit. Int. J. 12, 298–308. 10.1016/S1053-8100(02)00072-7 12763010

[B41] Seli P. Carriere J. S. Wammes J. D. Risko E. F. Schacter D. L. Smilek D. (2018). On the clock: evidence for the rapid and strategic modulation of mind wandering. Psychol. Sci. 29, 1247–1256. 10.1177/0956797618761039 29547349

[B42] Seli P. Smilek D. Schacter D. L. (2016). Mind-wandering with and without intention. Trends Cognit. Sci. 20, 605–617. 10.1016/j.tics.2016.05.010 27318437 PMC5004739

[B43] Smallwood J. (2013). Distinguishing how from why the mind wanders: a process–occurrence framework for self-generated mental activity. Psychol. Bullet. 139:519. 10.1037/a0030010 23607430

[B44] Smallwood J. Karapanagiotidis T. Ruby F. Medea B. Caso I. de Konishi M.. (2016). Representing representation: integration between the temporal lobe and the posterior cingulate influences the content and form of spontaneous thought. PLoS ONE 11:e0152272. 10.1371/journal.pone.0152272 27045292 PMC4821638

[B45] Sodré M. E. Wießner I. Irfan M. Schenck C. H. Mota-Rolim S. A. (2023). Awake or sleeping? Maybe both… A review of sleep-related dissociative states. J. Clin. Med. 12:3876. 10.3390/jcm12123876 37373570 PMC10299622

[B46] Tagliazucchi E. Laufs H. (2014). Decoding wakefulness levels from typical fMRI Resting-state data reveals reliable drifts between wakefulness and sleep. Neuron 82, 695–708. 10.1016/j.neuron.2014.03.020 24811386

[B47] Thompson B. Daniel L. G. (1996). Factor analytic evidence for the construct validity of scores: a historical overview and some guidelines. Educ. Psychol. Measur. 56, 197–208. 10.1177/0013164496056002001

[B48] Topalidis P. Heib D. P. J. Baron S. Eigl E.-S. Hinterberger A. Schabus M. (2023). The virtual sleep lab—a novel method for accurate four-class sleep staging using heart-rate variability from low-cost wearables. Sensors 23:2390. 10.3390/s23052390 36904595 PMC10006886

[B49] van Rijn E. Eichenlaub J.-B. Lewis P. A. Walker M. P. Gaskell M. G. Malinowski J. E.. (2015). The dream-lag effect: selective processing of personally significant events during rapid eye movement sleep, but not during slow wave sleep. Neurobiol. Learning Memory 98–109. 10.1016/j.nlm.2015.01.009 25683202

[B50] Wamsley E. J. (2014). Dreaming and offline memory consolidation. Curr. Neurol. Neurosci. Rep. 14, 1–7. 10.1007/s11910-013-0433-5 24477388 PMC4704085

[B51] Wamsley E. J. Stickgold R. (2019). Dreaming of a learning task is associated with enhanced memory consolidation: Replication in an overnight sleep study. J. Sleep Res. 28:e12749. 10.1111/jsr.12749 30091247 PMC6338510

[B52] Wamsley E. J. Tucker M. Payne J. D. Benavides J. A. Stickgold R. (2010). Dreaming of a learning task is associated with enhanced sleep-dependent memory consolidation. Curr. Biol. 20, 850–855. 10.1016/j.cub.2010.03.027 20417102 PMC2869395

