# Supplementary material for: Targeted dream incubation at a distance: the development of a remote and sensor-free tool for incubating hypnagogic dreams and mind-wandering
Source: Front Sleep. 2024 May 28;3:1258345. doi: 10.3389/frsle.2024.1258345 (PMC12713799; doi:10.3389/frsle.2024.1258345)
Supplement: Supplementary file 1 [file Data_Sheet_1.DOCX]

**Supplementary Materials:**

**Supplementary Table A: Full Partial Correlations Matrix**

|  | **1** | **2** | **3** | **4** | **5** | **6** | **7** | **8** | **9** | **10** | **11** | **12** | **13** | **14** | **15** | **16** | **17** | **18** | **19** | **20** | **21** | **22** | **23** |
| --- | --- | --- | --- | --- | --- | --- | --- | --- | --- | --- | --- | --- | --- | --- | --- | --- | --- | --- | --- | --- | --- | --- | --- |
| **1. Age** | **—** | — | — | — | — | — | — | — | — | — | — | — | — | — | — | — | — | — | — | — | — | — | — |
| **2. Positive_Hyp_** | .02 | **—** | — | — | — | — | — | — | — | — | — | — | — | — | — | — | — | — | — | — | — | — | — |
| **3. Positive_FMT_** | -.14 | **.28*** | **—** | — | — | — | — | — | — | — | — | — | — | — | — | — | — | — | — | — | — | — | — |
| **4. Negative_Hyp_** | .16 | **-.66***** | **.36**** | **—** | — | — | — | — | — | — | — | — | — | — | — | — | — | — | — | — | — | — | — |
| **5. Negative_FMT_** | -.15 | .23 | **-.66***** | **.45***** | **—** | — | — | — | — | — | — | — | — | — | — | — | — | — | — | — | — | — | — |
| **6. Images_Hyp_** | **.28*** | .06 | .10 | .11 | .22 | **—** | — | — | — | — | — | — | — | — | — | — | — | — | — | — | — | — | — |
| **7. Images_FMT_** | **-.30*** | .05 | -.19 | .06 | -.26 | **.39**** | **—** | — | — | — | — | — | — | — | — | — | — | — | — | — | — | — | — |
| **8. Words_Hyp_** | .11 | .11 | .09 | -.11 | .13 | -.12 | .01 | **—** | — | — | — | — | — | — | — | — | — | — | — | — | — | — | — |
| **9. Words_FMT_** | -.04 | .00 | -.03 | .07 | -.01 | -.19 | -.10 | **.43***** | **—** | — | — | — | — | — | — | — | — | — | — | — | — | — | — |
| **10. Novel_Hyp_** | .08 | -.02 | .01 | -.10 | -.11 | .04 | .07 | .01 | .04 | **—** | — | — | — | — | — | — | — | — | — | — | — | — | — |
| **11. Novel_FMT_** | .09 | .13 | -.02 | .07 | -.01 | -.16 | .10 | .00 | -.04 | .22 | **—** | — | — | — | — | — | — | — | — | — | — | — | — |
| **12. Freely moving_Hyp_** | .06 | -.15 | .08 | -.11 | .26 | .06 | .02 | .04 | .06 | .19 | -.01 | **—** | — | — | — | — | — | — | — | — | — | — | — |
| **13. Freely moving_FMT_** | -.04 | .16 | -.01 | .12 | -.20 | .02 | .07 | .05 | -.13 | -.06 | .10 | **.38**** | **—** | — | — | — | — | — | — | — | — | — | — |
| **14. Meaningful_Hyp_** | -.15 | .24 | -.03 | .11 | .09 | -.16 | -.04 | .08 | -.22 | -.04 | -.03 | .14 | -.02 | **—** | — | — | — | — | — | — | — | — | — |
| **15. Meaningful_FMT_** | .16 | **-.28*** | .24 | **-.31*** | .08 | .16 | .07 | -.15 | **.44***** | .06 | .12 | -.02 | .10 | **.45***** | **—** | — | — | — | — | — | — | — | — |
| **16. Topical shifts_Hyp_** | -.19 | -.13 | -.05 | .12 | -.23 | .02 | -.14 | .19 | -.20 | .11 | .12 | .13 | -.20 | .14 | .10 | — | — | — | — | — | — | — | — |
| **17. Topical shifts_FMT_** | .09 | .17 | -.10 | .08 | .05 | -.11 | .06 | -.13 | .11 | .04 | **-.28*** | .07 | .17 | -.18 | .05 | **.40**** | — | — | — | — | — | — | — |
| **18. Current concerns_Hyp_** | -.01 | -.09 | -.10 | .23 | -.13 | .04 | -.06 | **.45***** | -.15 | -.04 | .01 | -.01 | .02 | -.04 | .12 | -.04 | -.01 | — | — | — | — | — | — |
| **19. Current concerns_FMT_** | -.05 | -.16 | -.06 | -.08 | .02 | .10 | -.04 | -.01 | **.26*** | .12 | .05 | -.24 | .21 | .25 | -.25 | .00 | **.27*** | .24 | — | — | — | — | — |
| **20. Bizarre_Hyp_** | -.06 | .09 | -.13 | **.26*** | -.14 | -.03 | .01 | .11 | -.17 | **.43***** | .05 | .09 | -.18 | -.18 | .19 | -.12 | .04 | -.07 | .04 | — | — | — | — |
| **21. Bizarre_FMT_** | -.06 | -.21 | .13 | **-.28*** | **.38**** | -.03 | .07 | -.06 | .17 | -.05 | .22 | -.18 | .18 | .12 | -.24 | .18 | .21 | -.05 | -.16 | **.51***** | — | — | — |
| **22. Emotional_Hyp_** | .11 | -.04 | -.07 | .06 | -.16 | .19 | .05 | .01 | .21 | .07 | **.33*** | -.02 | -.06 | **.51***** | **-.45***** | -.14 | .14 | .05 | -.17 | -.09 | -.01 | — | — |
| **23. Emotional_FMT_** | .02 | .21 | .04 | .14 | **.26*** | **-.37**** | .20 | -.11 | -.14 | -.06 | -.20 | .02 | .23 | **-.40**** | **.55***** | .16 | **-.27*** | .12 | .14 | .08 | .04 | **.54***** | — |
| **24. Number of dreams reported** | .03 | .12 | -.04 | .15 | -.04 | -.07 | .01 | -.16 | .14 | **.31*** | -.13 | -.11 | -.07 | .04 | -.11 | .02 | -.08 | .12 | -.13 | -.01 | -.03 | -.13 | .06 |

Hyp = hypnagogia, FMT = Freely moving thought; p < .05*; p < .01**, p < .001***

**
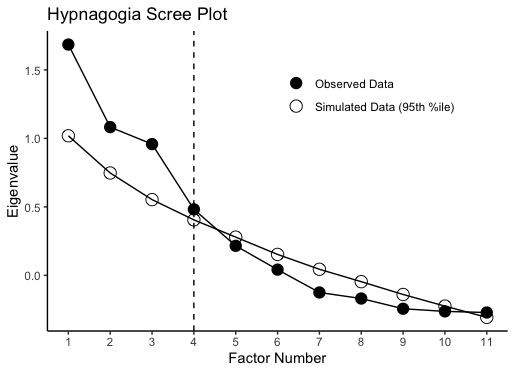
**

**Supplementary Figure S1:** Parallel analysis on hypnagogia data. Simulated data consisted of 1000 iterations from the same data structure as the observed data, with eigenvalues drawn via a common factors (maximum likelihood) model. Four factors were recommended, as observed data eigenvalues remained above the simulated data eigenvalues latest at the 4^th^ factor. Thus, when implementing the EFA for hypnagogia, we requested 4 distinct loadings.

**
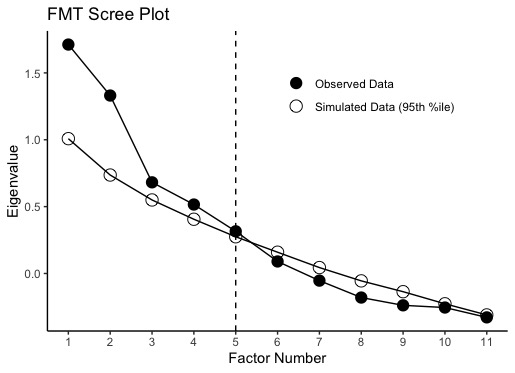
**

**Supplementary Figure S2:** Parallel analysis on FMT data. Simulated data consisted of 1000 iterations from the same data structure as the observed data, with eigenvalues drawn via a common factors (maximum likelihood) model. Five factors were recommended, as observed data eigenvalues remained above the simulated data eigenvalues latest at the 5^th^ factor. Thus, when implementing the EFA for FMT, we requested 5 distinct loadings.
